# Assessment of Novel Routes of Biomethane Utilization in a Life Cycle Perspective

**DOI:** 10.3389/fbioe.2016.00089

**Published:** 2016-12-19

**Authors:** Elham Ahmadi Moghaddam, Serina Ahlgren, Åke Nordberg

**Affiliations:** ^1^Department of Energy and Technology, Swedish University of Agricultural Sciences (SLU), Uppsala, Sweden

**Keywords:** biomethane, methanol, DME, ammonia, energy balance, environmental impacts

## Abstract

Biomethane, as a replacement for natural gas, reduces the use of fossil-based sources and supports the intended change from fossil to bio-based industry. The study assessed different biomethane utilization routes for production of methanol, dimethyl ether (DME), and ammonia, as fuel or platform chemicals and combined heat and power (CHP). Energy efficiency and environmental impacts of the different pathways was studied in a life cycle perspective covering the technical system from biomass production to the end product. Among the routes studied, CHP had the highest energy balance and least environmental impact. DME and methanol performed competently in energy balance and environmental impacts in comparison with the ammonia route. DME had the highest total energy output, as fuel, heat, and steam, among the different routes studied. Substituting the bio-based routes for fossil-based alternatives would give a considerable reduction in environmental impacts such as global warming potential and acidification potential for all routes studied, especially CHP, DME, and methanol. Eutrophication potential was mainly a result of biomass and biomethane production, with marginal differences between the different routes.

## Introduction

Biogas [composed mainly of methane (CH_4_) and carbon dioxide (CO_2_)] produced from waste, residues, and energy crops through anaerobic digestion (AD) is a versatile renewable energy source that can be used for replacement of fossil fuels in combined heat and power (CHP) production or as a vehicle fuel after upgrading (removal of carbon dioxide). Upgraded biogas (biomethane) can also be injected to the gas grid, replacing natural gas as a feedstock for producing chemicals and materials (Weiland, 2010). The production of biogas through AD has been rated as an energy-efficient and environmentally beneficial technology for renewable energy production (Fehrenbach et al., 2008). Furthermore, use of the digestate as an organic fertilizer can reduce dependence on energy-intensive mineral fertilizers, further mitigating greenhouse gas (GHG) emissions (Pöschl et al., 2010).

Anaerobic digestion has previously mainly been associated with the treatment of animal manure and sewage sludge. However, the limited production rate and methane yield of these feedstocks has led to the introduction of energy-rich co-feedstocks in order to increase biogas production. Among high-yielding co-feedstocks, energy crops are important. In Germany, an estimated 16% of agricultural land was used for energy crops in 2011, of which 40% were energy crops for AD (FNR, 2012). Maize and sugar beet have the highest gross energy potential of commonly grown energy crops (Weiland, 2010). In addition, the cultivation of these crops produces the lowest specific GHG emissions of all energy crops (Hartmann, 2006; Börjesson et al., 2015). Maize cultivation is increasing within northern Europe due to the temperate climate, aided by the global warming effect, creating a need to manage this crop and its residues (Pöschl et al., 2010; Menardo et al., 2015).

Combined heat and power systems are a common utilization pathway for biogas and a promising method for the industrial sector to improve its carbon credentials without changing its fuel or heat demand. The total electricity and heat utilized from biogas-based CHP plants in the European Union in 2013 was 34 TWh electricity and 4.4 TWh heat (Biogas Barometer, 2014). However, the thermal efficiency of large-scale CHP units is in the range of 40–50%, so it is clear that only a part of the heat produced is utilized, i.e., a large proportion of the heat from CHP production is wasted. Thus, lack of local heat sinks reduces the total efficiency of biogas use. Furthermore, the use of upgraded biogas as a vehicle fuel can be limited by a lack of gas infrastructure, complicating storage, and distribution. Biofuels for transportation is strongly regulated (e.g., in the EU Renewable Energy Directive) and has a high degree of political dependency, i.e., politicians decide the rules on tax exemption for biofuels, which is vital for the economic returns. Moreover, the share of biofuels within transportation originating from food crop-based feedstock is being capped, since politicians fear that increasing production of biofuel crops can lead to displacement of food crop production and cultivation of virgin arable land (Ahlgren and Di Lucia, 2014). In this perspective, it is interesting to explore new options for utilization of biogas, i.e., not just for CHP production or biomethane in the gas phase for vehicle use but also as a source for producing more high-value biofuels or platform chemicals.

Biomethane has similar properties to natural gas and has the potential to produce platform chemicals conventionally derived from natural gas converted to syngas, i.e., a mixture of carbon monoxide (CO) and hydrogen gas (H_2_), with subsequent catalytic synthesis. Synthesis of methanol is one such option. A methanol economy has been suggested for the future, in which methanol replaces fossil fuels as a means of energy storage, ground transportation fuel, and raw material for synthetic hydrocarbons and their products (United States Federal Transit Administration, 1998). Methanol, as a liquid material, can be easily stored, transported, and used. A large variety of chemicals are already produced from methanol products, such as gasoline, ethylene, and propylene, which are the most widely produced chemicals by the petrochemical industry (Gill et al., 2011) and the building blocks of many essential polymers. As an alcohol-based fuel, methanol has efficient combustion and distribution properties, which can be exploited directly as fuel or blended with petrol, converted to dimethyl ether (DME) as a diesel replacement, used in the biodiesel production process, or even used in a direct methanol fuel cell (DMFC) (Demirbas, 2005; Olah, 2005; Liu et al., 2007).

Dimethyl ether is the simplest ether, primarily produced directly from syngas or indirectly by dehydration of methanol. It is used as a propellant for aerosol products, a refrigerant, an extraction agent, and a fuel for welding and transportation. The fuel characteristics of DME are similar to those of liquefied petroleum gas (LPG), which is stored under pressure and can use the same existing infrastructure as LPG (Semelsberger et al., 2006). Moreover, DME can be used in gas turbines and fuel cells for electricity generation. Combustion of DME does not produce soot, and it is considered a clean fuel, with limited levels of particulate matter (PM) and nitrous oxide (NO_x_) emissions (Yamada, 2003). Previous studies by our research group have shown that methanol and DME fuels produced from biogas have a relatively low primary energy (PE) input and GHG emissions and high energy efficiency (Moghaddam et al., 2015).

Ammonia is another platform chemical serving as the building block of many chemical products and also as a precursor to nitrogen fertilizers, which significantly contribute to crop yield and the nutritional requirements of living organisms (Makhlouf et al., 2015). Ammonia is synthesized in the Haber–Bosch process, which is a highly energy-demanding process leading to large amounts of GHG emissions. Many studies have been devoted to energy demand and GHG emissions from the ammonia industry [e.g., Bouwman et al. (1997), Rafiqul et al. (2005), and Zhou et al. (2010)]. The estimated average energy use in the ammonia industry in Europe is 34.7 GJ/ton ammonia (De Haas and Van Dijk, 2010). Worldwide, the nitrogen fertilizer industry consumes about 1.2% of global PE consumption, of which more than 90% is used in the production of ammonia (Tunå et al., 2014). Moreover, the GHG emissions related to fertilizer production are increasing, in pace with efforts to secure a sustainable supply of food for the growing global population (Tilman et al., 2011).

When novel conversion routes are suggested, it is important to assess the energetic and environmental performance of these in a systems perspective and compare it with that of conventional techniques. Life cycle assessment (LCA) is an accepted method for analyzing the environmental performance of products or services, as it not only improves understanding of how alternative systems compare with each other but also how different sub-processes in a system affect the overall results (Baumann and Tillman, 2004).

The objective of this study was to assess the environmental impact and energy balance of utilizing biogas from AD of maize for CHP production, and for production of methanol, DME, and ammonia as alternative routes, in a Swedish perspective. Sweden has invested in many AD plants, which upgrade biogas to vehicle fuel. However, as previously mentioned, there are problems related to storage and distribution of compressed biogas. Therefore, there is great interest in studying alternative utilization of biogas.

In a previous study (Moghaddam et al., 2015), we assessed the energy efficiency and global warming potential (GWP) of conversion of biogas to different fuels, covering the technical system from raw biogas to use in city buses. In the present study, the scope was expanded to cover the agricultural system for production of energy crops (maize), the AD process, and the conversion to different chemicals or fuels. In this analysis, eutrophication potential (EP) and acidification potential (AP) were included, as several studies have shown that biogas from energy crops could potentially have higher impacts than fossil fuel-based systems due to nitrate ${\text{(NO}}_{3}^{-}\text{)}$ and phosphate ${\text{(PO}}_{4}^{3-}\text{)}$ leaching from fertilized soil (ADEME, 2011; Labutong, 2012; Rehl et al., 2012).

The novel aspects of the work compared with previous studies are:

- Assessment of impacts associated with production of methanol, DME, and ammonia as platform chemicals generated from biogas using maize crops, in comparison with utilizing the biogas for CHP production.
- Comparison of the environmental impacts of the bio-based products (methanol, DME, ammonia, and CHP) with those of their fossil-based alternatives.

## Materials and Methods

The energy inputs and environmental performance of biomass (maize) production (including all crop cultivation activities and fertilizer production), road transport of feedstock and digestate, digestate processing and handling, biomethane production (including ensiling, AD, upgrading, and injection to the gas grid), and biomethane utilization through different routes of CHP, methanol, DME, and ammonia production were included in the analysis. The assessment was carried out in accordance with the ISO, 14040/44 methodology for LCA (ISO, 2006a,b). An attributional LCA approach was used to model the inventory of the life cycle, which aimed to describe the environmentally relevant physical flows to and from the life cycle and its subsystems. This can be compared with consequential LCA modeling, which examines the environmental consequences of marginal changes in a life cycle, often with a market-oriented approach (Zamagni et al., 2012). The present study included replacement of fossil products on the market, which is normally covered in consequential LCA modeling, but did not assess the marginal market effects.

The energy balance was evaluated as the ratio between output energy from the systems and the PE input. The PE input was calculated as the energy input to the system boundary, and therefore internal use of energy (i.e., heat recirculation, burning part of the biogas as process fuel) was not included as an energy input. Included in PE inputs were energy for cultivation, transportation, and conversion. Furthermore, energy for production of energy inputs (e.g., extraction of fossil fuels, conversion, transmission, and distribution losses) were included (Energimyndigheten, 2006). Factors used for conversion of data on electricity and of diesel to PE are presented in Table 1. The PE factor was defined as the ratio between PE and delivered useful energy.

**Table 1:** **Primary energy (PE) factor for different energy carriers (MJ/MJ energy carrier).^a^**

Energy carrier	Specification	Primary energy factor
Electricity	Nordic electricity mix (NORDEL)	2.01
Fuel	Diesel, low-sulfur	1.35

*^a^Ecoinvent (2015) ver.3-2*.

The environmental impacts assessed included GWP, EP, and AP. GWP is defined as the contribution to atmospheric absorption of infrared radiation by anthropogenic derived gases, such as CO_2_, CH_4_, and N_2_O, during the life cycle of the product in each scenario, calculated as CO_2_-equivalents (CO_2_-eq), see Table 2. In the present study, GWP refers to a time horizon of 100 years based on Myhre (2013). Biogenic carbon was considered carbon neutral and thus not included in the GHG accounting.

**Table 2:** **Equivalency factors used in the study**.

	Global warming potential (GWP)^a^	Eutrophication potential (EP)^b^	Acidification potential (AP)^c^
CO_2_ (fossil)	1		
CH_4_	28		
N_2_O	265		
NO_x_		0.13	0.7
NH_3_		0.35	1.88
$PO_{4}^{3-}$		1	
SO_2_			1
Total nitrogen (water)		0.42	
Total phosphorus (water)		3.07	

*^a^Global warming potential, expressed as carbon dioxide equivalents. Data taken from Myhre (2013)*.

*^b^Eutrophication potential, expressed as phosphate equivalents. Data taken from Clark and Macquarrie (2008)*.

*^c^Acidification potential, expressed as sulfur dioxide equivalents. Data taken from Bouman et al. (1999)*.

Eutrophication potential is calculated as ${\text{PO}}_{4}^{-3}$-equivalents (${\text{PO}}_{4}^{-3}$-eq) using equivalency factors (Table 2). According to this, an increased input of nutrients to aquatic systems leads to increased generation of biomass, which through aerobic decomposition results in oxygen depletion in water ecosystems and serious damage to biological populations. Furthermore, EP takes into consideration that when nitrogen compounds are emitted to air, a fraction can reach aquatic systems by deposition. AP is calculated as the amount of protons released in a terrestrial system [including nitrogen compounds and sulfur dioxide (SO_2_)], and is calculated as SO_2_-equivalents (SO_2_-eq) using equivalency factors (Table 2).

The different environmental impact mitigation prospects for the implementation of bio-based products considering a complete substitution of fossil substitutes (alternatives) were quantified. The fossil alternatives for the main products and the by-products (heat and steam) were based on natural gas conversion. The net emissions in terms of GWP, EP, and AP for the different products were calculated based on the differences in emissions between the biomethane-based production routes and their fossil-based alternatives, on a functional unit (FU) basis. Data for the fossil-based products were sourced from Ecoinvent database v3.2 (Ecoinvent, 2015).

### Functional Unit and System Boundaries

In order to compare different scenarios, a common basis for calculation had to be defined. The aim of this study was to assess alternative routes for utilizing biogas from AD of maize. For this type of research question, an input-based FU such as 1 ha or 1 ton of biomass is usually appropriate (Ahlgren et al., 2015). In this study, we also want to include the agricultural production systems, thus, the FU was defined as 1 ha land cultivated with maize during 1 year.

**Figure 1:**
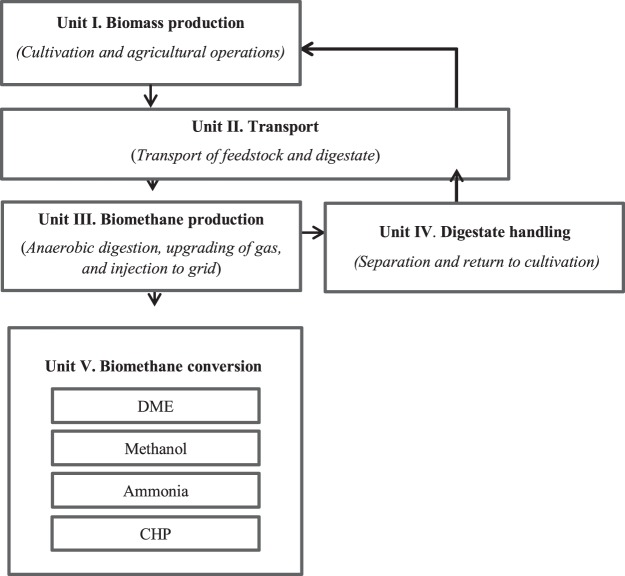
**Description of the systems studied**.

Figure 1 shows a graphical description of the current study, encompassing: agricultural operations for biomass production (unit I), transportation of feedstock and digestate (unit II), biogas plant operations and injection of biomethane to the grid (unit III), digestate handling (unit IV), and the energy conversion technologies for CHP, methanol, DME, and ammonia production (unit V). The *biomass-to-biomethane* stage, including units I to IV, was assumed to be identical for all scenarios assessed, and therefore this analysis was included for all scenarios. Production of capital goods such as machinery and buildings was not included in the calculations, as it was estimated that this would have only slight effects on the overall results (Forster et al., 2007).

## System Description

The assessment was based on an existing biogas production plant producing 100 GWh/year, which is similar to Sweden’s largest AD facility. The upgraded gas was assumed to be injected to the regional gas grid and utilized at another location. The scale of the utilization scenarios was chosen to match the amount of methane produced. This meant that the chemical and fuel production was rather small scale compared with existing fossil-based commercial plants.

### Biomass Production and Transportation

Production of maize feedstock comprises cultivation, application of mineral fertilizer, pesticide, and digestate, harvesting, and in-field transport (~2 km). The main energy inputs and emissions are related to diesel use and combustion during harvest and cultivation operations by agricultural machinery. In this study, the AD produced enough digestate to considerably reduce the use of mineral fertilizer for maize cultivation, while the remaining area was fertilized with NPK fertilizer. Biogenic emissions of N_2_O were estimated according to IPCC (2006) to be 967 kg CO_2_-eq/FU. Emissions related to nutrient leakage from application of the digestate and fertilizers were estimated according to Börjesson and Tufvesson (2011) to be 16 kg ${\text{PO}}_{4}^{3-}$ eq/FU. The transport distance of harvested material (feedstock) from field to AD plant was set to 20 km, based on Nilsson (1995). Ensiling of harvested material was assumed to be carried out at the AD plant. Characteristics of maize as an energy crop and digestate assumed in the study are presented in Table 3. Details (inventory data) of maize cultivation and transport to the AD plant are presented in Table S1 in Supplementary Material.

**Table 3:** **Characteristics of maize as an energy crop and digestate assumed in the study (ww = wet weight)**.

Maize yield	ton ww/ha/year	43
Dry matter (DM) concentration	%	30
Methane yield	Nm^3^/ton DM	316
Biomethane yield	GJ/ha/year	143^a^
Digestate yield	ton/ha/year	34.4

*^a^Gross production of biogas including internal use; net biogas production is 130 GJ/ha/year*.

### Biogas Production and Upgrading

The biogas plant was assumed to have a biomethane production capacity of 10 million Nm^3^/year (1 atm, 0°C), and an annual feedstock input of 108,000 ton maize silage from 2,512 ha arable land. At the biogas plant, ensiling is predominantly carried out for storage reasons (Pakarinen et al., 2011). The maize silage is delivered to an on-site hopper and mixer. In order to obtain a feedstock with a viscosity suitable for the mixing equipment, a share of the liquid fraction after solid–liquid separation of the digestate is returned to the intake of feedstock (Rehl et al., 2012; Meixner et al., 2015). The feedstock then passes through a macerator before being pumped into the anaerobic digester, operated as a wet fermentation process under mesophilic conditions, to produce 23,400 Nm^3^ of biomethane per day in the present case. Based on the daily consumption of 296 ton wet weight feedstock, this is equivalent to a biomethane yield of 79 Nm^3^/ton wet weight feedstock. The raw biogas was assumed to have a composition of 55% CH_4_. The heat requirement of the digester is met by burning part of the biogas and the electricity input for operating the AD plant is provided from the grid. Raw biogas is then upgraded in a water scrubber to natural gas quality (97% CH_4_). Based on Berglund and Börjesson (2006), the loss of CH_4_ was set to 1% of the biogas from the AD process and 2% from the upgrading process. Upgraded biogas (biomethane) was assumed here to be injected to the regional gas grid for further transformation in a CHP unit or a syngas production unit for methanol, DME, and ammonia production.

### Digestate Handling

The AD plant assumed in this case has an annual digestate output of 86,000 ton. The digestate generated is phase-separated using a screw press, which results in a solid and a liquid digestate. The annual output of liquid digestate is 77,400 ton and of solid digestate is 8,600 ton. The liquid digestate not used for improving the mix of feedstock substrate is stored in covered lagoons in order to reduce emissions of ammonia (NH_3_) and methane (Whiting and Azapagic, 2014), before it is transported to the field (20 km) for utilization as an organic fertilizer. The liquid digestate is applied using shallow injection in order to reduce the nitrogen loss as ammonia. The solid fraction is collected in containers and transported to farms, where it is stored in piles before application as a solid organic fertilizer on fields using broadcasting.

### Description of Different Routes of Biomethane Use

#### CHP Scenario

The CHP system studied comprised a combustion (gas) turbine with an installed electricity capacity of 9,500 kW and heat generation of 8,100 kW_th_ with a total efficiency of 90%. This capacity is over-dimensioned in relation to the amount of biomethane produced. However, the conversion efficiency of such a unit was used for the calculations. Output electricity is sold to the grid and the heat to district heating networks. The GHG emissions from a CHP plant are mainly due to carbon dioxide emissions from fuel combustion (Goehner et al., 2013), which were considered climate neutral in this study as they are of biogenic origin.

#### Syngas Production Unit

**Figure 2:**
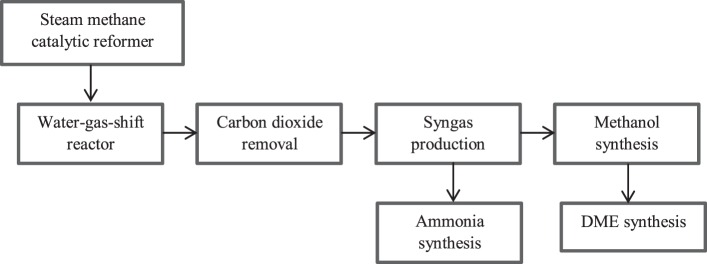
**Biomethane utilization route showing the syngas unit and the different units of methanol, DME, and ammonia synthesis**.

Biomethane was assumed to be converted to syngas in the methanol, DME, and ammonia routes (Figure 2). The syngas unit has been modeled in our previous work (Moghaddam et al., 2015). Syngas is produced from biomethane *via* steam reforming (Figure 2). The steam reformer is externally heated by burning part of the feed. The burner was modeled here as a combustion reactor in the Aspen Plus set-up to provide the energy required in the steam reformer. The syngas produced contains H_2_, CO, CO_2_, nitrogen gas (N_2_), and water vapor (H_2_O).

In order to achieve maximum H_2_ content in the gas, water–gas shift reactors are required, where CO is shifted with H_2_O to H_2_ in the water–gas shift reaction as an equilibrium reaction (Eq. 1). The systems are heat integrated using pinch technology. There is therefore energy conservation within the system boundary, such that the exported values of steam and heat are the lowest that can be achieved with good engineering. The syngas produced was guided to the methanol, DME, or ammonia production route. In all cases, the steam generated was medium pressure steam at 26 bar and 275°C. The water produced was assumed to be at 130°C, which primarily satisfied the internal heat requirement and was hence compatible with district heating (Moghaddam et al., 2015). Electricity input to the syngas process is provided from the grid.

(1) $${\text{CO + H}}_{\text{2}}\text{O}\to {\text{CO}}_{\text{2}}{\text{ + H}}_{2}$$

#### Methanol Production Scenario

The methanol reaction is described in Eq. 2. The methanol reactor was simulated using a steam-raising type of reactor based on micro-channel technology that is cooled by boiling feed water. The product stream is condensed and methanol and water are removed. The gas is recycled to the inlet of the reactor except for a small part that is removed as tail gas. Methanol and water are distilled to yield 99.9% pure methanol (Lundgren et al., 2013). Output from 1 MW biogas is 0.75 MW methanol. Thus, per ton feedstock, the fuel plant generates 113.5 kg methanol. Energy input and emissions data for methanol production were based on our previous work (Moghaddam et al., 2015).

(2) $${\text{2H}}_{\text{2}}\text{ + CO}\to {\text{CH}}_{\text{3}}\text{OH}$$

#### DME Production Scenario

The methanol synthesis described above was considered an input to DME synthesis. Methanol is dehydrated in the presence of a catalyst, resulting in the production of DME as described in Eq. 3.

(3) $${\text{2CH}}_{\text{3}}\text{OH}\to {\text{CH}}_{\text{3}}{\text{OCH}}_{\text{3}}{\text{ + H}}_{\text{2}}\text{O}$$

The product is cooled and methanol, DME, and water are separated in a two-step distillation process. Methanol is recycled back to the reactor inlet. The purge stream contains methanol, DME, water, and trace amounts of H_2_, CO, etc. The purge stream is burnt to produce heat and power. The present simulations were based on pressurized storage of DME in a vacuum-insulated vessel requiring no energy for storage (Hansen and Mikkelsen, 2001). This form of storage was chosen because the DME is separated in the liquid phase under pressure, and therefore keeping it under pressure is the most efficient storage method for the system (Moghaddam et al., 2015). Output from 1 MW biogas is 0.90 MW DME fuel. Thus per ton of wet weight feedstock, the fuel plant generates 94 kg DME fuel. Energy input and emissions data for DME production were based on our previous work (Moghaddam et al., 2015).

#### Ammonia Production Scenario

Assumptions about the ammonia plant were based on work performed by Tunå et al. (2014). In this route, the syngas produced through steam reforming is compressed and directed to an ammonia synthesis unit, where the hydrogen is catalytically reacted with nitrogen derived from process air to form anhydrous liquid ammonia in what is known as the Haber–Bosch process (described in Eq. 4).

(4) $${\text{3H}}_{\text{2}}{\text{ + N}}_{\text{2}}\to {\text{ 2NH}}_{\text{3}}$$

The ammonia synthesis reaction is an exothermic reaction in the presence of an iron catalyst at high pressure (100–250 bar) and temperature (350–550°C). In the present study, it was assumed that the heat released was used in the district heating grid. The conversion to ammonia in the synthesis is low (20–30%), but the unreacted gases are recirculated. The final product is refrigerated and stored at low pressure (Ahlgren et al., 2008). The ammonia plant generates 79 kg NH_3_-N per ton wet weight feedstock.

## Results

Inventory data for the biomass-to-biomethane production (units I–IV) and different routes of biomethane use (unit V), based on the FU are presented in Tables S1 and S2 in Supplementary Material. This section presents the results for each impact category in turn, with unit processes as described in Figure 1.

### Primary Energy Input

**Figure 3:**
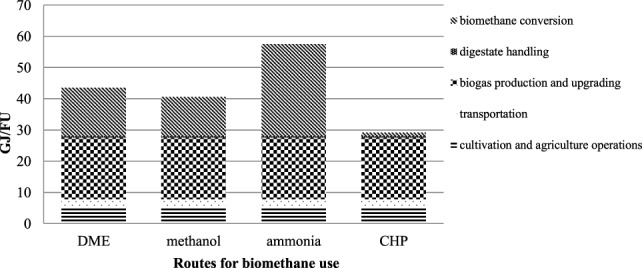
**Primary energy input to the biomass-to-biomethane production chain and the different routes of biomethane use**.

The results for PE use are presented in Figure 3. Biogas production and upgrading (unit III) was the largest energy consumer (20 GJ per FU), accounting for more than 71% of the total energy input to the biomass-to-biomethane chain. Electricity (4.3 GJ per FU) and heat demand (13.7 GJ per FU) for the AD plant corresponded to 3 and 9.6% of the proportion of biogas produced, respectively. Heat for the AD plant was provided by burning part of the biogas, which reduced the net biogas output to 130 GJ per FU. Energy input as electricity for the upgrading of raw biogas in the water scrubber was 11 GJ/FU. This high electricity demand is due to the high pressure used in the water scrubber, resulting in increased solubility of carbon dioxide in water (Benjaminsson and Nilsson, 2009). Maize cultivation (unit I) and transportation (unit II) contributed 12 and 7%, respectively, of the total PE input to the biomass-to-biomethane production chain.

Among the routes of biomethane use, the ammonia production process had the highest PE input (29 GJ/FU). This was mainly related to the syngas production unit Figure 2. The DME and methanol production processes had an approximate energy requirement of 15.5 and 12.6 GJ/FU, respectively. This energy demand was related to the electricity required for converting biomethane to syngas. The CHP production process had the lowest energy requirement (1 GJ/FU), which was mainly related to operating equipment.

#### Energy Output and Energy Balance

In the system studied, which included the biomass-to-biomethane chain and the different biomethane utilization routes, the output consisted of a main product (methanol, DME, ammonia, or electricity) and by-products (heat and steam). The methanol and DME production processes both yield heat and steam as by-products. The steam is of sufficient quality to be sold for use in other production processes, while the heat is of sufficient quality to be sold as district heating. In the present study, the high pressure steam produced in the ammonia route was recycled in the system and no steam export was considered. However, the ammonia route generates a large amount of heat for district heating as a by-product to the main chemical product. Electricity was considered the main product for the CHP scenario, with district heating as a co-product. The different energy inputs, outputs, and energy balance of the routes for biomethane use studied here are presented in Table 4. The CHP scenario had the best energy performance (4.00), followed by DME and methanol, which both had a high energy balance (3.04 and 2.85, respectively), whereas the energy balance for ammonia was low (1.76) (Table 4). Ammonia had the highest energy input (58 GJ/FU), whereas DME and methanol had a total input of 44 and 41 GJ/FU, respectively.

**Table 4:** **Amount of product, heat, and steam (GJ/FU) produced in the different scenarios**.

	Input (GJ/FU)	Output (GJ/FU)				Energy balance (out/in)
	Total	Product	Heat	Steam	Total	
DME	44	116	10	8	134	3.04
Methanol	41	98	9	11	117	2.85
Ammonia	58	63	39	0	102	1.76
CHP	29	63	53	0	116	4.00

### Global Warming Potential

**Figure 4:**
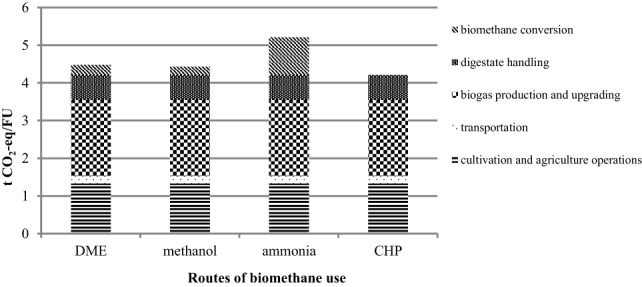
**Global warming potential of the biomass-to-biomethane production chain and the different routes of biomethane use**.

As shown in Figure 4, the total GWP of the biomass-to-biomethane production chain was assessed to be 4.2 ton CO_2_-eq/FU. Biogas production and upgrading (unit III) was the largest contributor to GHG emissions (2 ton CO_2_-eq/FU), mainly as methane emissions (1.7 ton CO_2_-eq/FU) and energy use for the AD plant and the water scrubber of the upgrading unit. Based on Berglund and Börjesson (2006), CH_4_ losses correspond to 1% of the biogas from AD and 2% of the biogas from upgrading processes, which in this study comprised 0.7 and 1 ton CO_2_-eq/FU, respectively.

Cultivation operations for biomass production (1.3 ton CO_2_-eq/FU) accounted for 31% of the total GWP from the biomass-to-biomethane production chain. The main contribution in the biomass production step (unit I) was the high biogenic emissions from soil, which included direct and indirect N_2_O emissions and contributed 73% of the GWP of the biomass production step. Emissions from agricultural machinery contributed 19% of the GWP of the biomass production step. Fertilizer production contributed approximately 6% of the GWP from the biomass production stage (unit I).

Among the biomethane use routes, ammonia had the highest level of GWP, 1 ton CO_2_-eq/FU. GHG emissions from the ammonia plant are mainly related to energy use in the gas reforming unit. The DME and methanol routes had approximately similar levels of GWP (0.26 and 0.21 ton CO_2_-eq/FU), respectively, mainly through emissions related to energy use by the syngas reformer. The GWP from the CHP unit was negligible because the CO_2_ was considered carbon neutral.

### Eutrophication and Acidification

**Figure 5:**
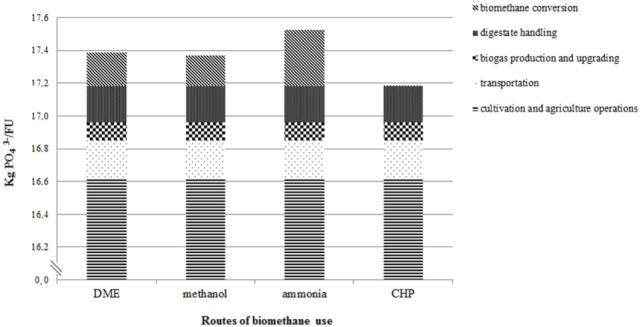
**Eutrophication potential of the biomass-to-biomethane production chain and the different routes of biomethane use**.

Cultivation operations for biomass production (unit I) were the main contributor to EP, accounting for 16.6 kg ${\text{PO}}_{4}^{3-}$/FU, which represented 97% of the total EP related to the biomass-to-biomethane production chain (Figure 5). This included leaching of ${\text{PO}}_{4}^{3-}$ and ${\text{NO}}_{3}^{-}$ to water, emissions of NH_3_ to the air from cultivation, and emissions of NO_x_ and NH_3_ from diesel combustion. Among the routes of biomethane use, ammonia production had the highest EP (0.34 kg ${\text{PO}}_{4}^{3-}$ eq/FU). Methanol and DME were in the same range (0.19 and 0.21 kg ${\text{PO}}_{4}^{3-}$ eq/FU, respectively). The CHP scenario had no significant eutrophication effects.

**Figure 6:**
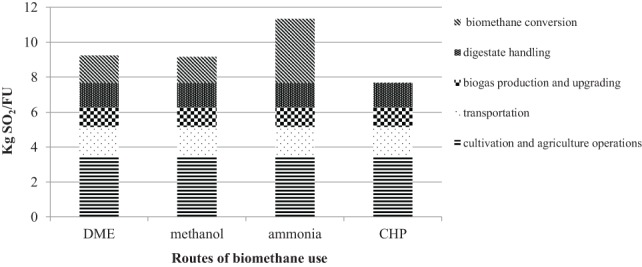
**Acidification potential of the biomass-to-biomethane production chain and the different routes of biomethane use**.

Cultivation operations (unit I) had the highest emissions related to AP, accounting for 3.41 kg SO_2_-eq/FU, which were mainly due to NO_x_ emissions related to diesel combustion and diesel production (Figure 6). Among the different routes for biomethane use, ammonia had the highest impact on AP (3.7 kg SO_2_-eq/FU), mainly related to high levels of NO_x_ and SO_2_ emissions from the production process. Methanol and DME were in the same range (1.49 and 1.56 kg SO_2_-eq/FU, respectively). The CHP scenario had no significant acidification effects.

### Replacement of Fossil Alternatives

Comparing the different bio-based routes on an FU basis, the base CHP production scenario can be considered the best option regarding energy balance and environmental impact. Among the novel routes, the methanol and DME production processes performed better than the ammonia route regarding energy balance and environmental impacts.

However, since we chose an input-based FU (1 ha of maize cultivation per year) for comparison of the bio-based routes, differences in product output were not considered. Therefore, we added the potential of the different products to replace fossil alternatives in the different routes (Table 5). In this way, we obtained a better comparison of the entire environmental impact of the systems. When comparing the different bio-based routes and their product outputs with replacement of the corresponding fossil products in terms of net emissions, it is clear that all the bio-based routes contributed a higher EP (16–17 kg ${\text{PO}}_{4}^{3-}$ eq/FU) due to the biomass-to-biomethane part of the system (Table 5). However, in comparison with the fossil alternatives, all the bio-based routes showed a lower AP, with DME giving the greatest reduction (−30 kg SO_2_-eq/FU) and ammonia the smallest (−6 kg SO_2_-eq/FU).

**Table 5:** **Total environmental impacts from the different routes studied and from the fossil substitute, and net emissions for the routes**.

	GWP (ton CO_2_-eq/FU)				EP (kg $PO_{4}^{3-}$-eq/FU)				AP (kg SO_2_-eq/FU)			
	DME	Methanol	Ammonia	CHP	DME	Methanol	Ammonia	CHP	DME	Methanol	Ammonia	CHP
**Biomethane route**												
Total environmental impacts from studied routes	4.5 (4.7)^a^	4.4 (4.6)^a^	5.2	4.2	17.4	17.4	17.5	17.2	9.2	9.2	11.3	7.7
**Fossil replacement**												
Fuel/chemical/electricity	4.7	2.6	6.7	9.4	1.1	0.5	0.8	0.7	37.0	24.7	14.7	24.9
Heat	0.3	0.2	0.8	1.5	n	n	0.1	0.1	0.8	0.7	3.0	4.1
Steam	0.1	0.2	n	n	n	n	n	n	1.3	1.8	n	n
Total, fossil replacement	5.1 (12)^b^	3.0 (9.9)^b^	7.5	10.9	1.1	0.5	0.9	0.8	39.1	27.2	17.7	29.0
Net emissions	−0.6 (−7.3)	1.4 (−5.3)	−2.3	−6.7	16.3	16.9	16.6	16.4	−29.9	−18.0	−6.4	−21.3

*Figures in brackets include combustion of the fuels*.

*n, not available*.

*^a^Emissions related to combustion of bio-based DME and methanol, comprising 0.2 ton CO_2_-eq/FU*.

*^b^Emissions related to fossil-based DME and methanol, comprising 6.9 ton CO_2_-eq/FU, respectively*.

Regarding the GWP, the highest reduction among the bio-based routes was found for the base CHP production scenario (−7 ton CO_2_-eq/FU). However, this comparison can be questioned, since the use phase of the bio-based products was not included. In the case of ammonia and CHP, the climate emissions are mainly related to the production phase. For the fuel cases, on the other hand, the climate emissions are mainly related to the use phase. Therefore, we also added the use of methanol and DME for comparison (shown in brackets in Table 5). By including the combustion of methanol and DME there is a considerable reduction in the GWP from the bio-based routes in comparison with the fossil alternatives, the net emissions amounting to −7.3 ton CO_2_-eq/FU and −5.3 ton CO_2_-eq/FU for DME and methanol, respectively.

In total, the methanol and DME routes had lower GWP than the ammonia route, even when combustion of methanol and DME was included (Table 5). Moreover, the AP was lower for methanol and DME than for ammonia. There was no significant difference in EP between the different bio-based routes, because this impact category is mainly related to the common biomass-to-biomethane part of the system (units I–IV).

### Sensitivity Analysis

In studies assessing novel technical routes, it is important to determine the impact of uncertainties on results and potential decisions. In this study, a sensitivity analysis was performed to evaluate the influence of changing the choice of electricity mix, the N_2_O emissions from soil, and the reduction of methane emissions from the upgrading of biogas on the results from the different bio-based routes, expressed per FU.

The choice of electricity mix in LCA calculations is often an uncertainty, and was also addressed in the sensitivity analysis. In the base case, an electricity mix for the Nordic countries (NORDEL) based on 35% hydropower, 11% biomass, 32% nuclear, 20% fossil, and 2% wind, solar, and geothermal sources was used for the calculations. The effect on the results of instead choosing a hard coal electricity mix or a Swedish electricity mix for the production chain was evaluated. The hard coal NORDEL was chosen to represent a fossil-based electricity mix with 98% hard coal, while the Swedish electricity mix is mainly based on nuclear energy (>50%) (Ecoinvent, 2015). The PE factors and GWP, EP, and AP factors used in the sensitivity analysis for the hard coal NORDEL and Swedish electricity mix are presented and compared with the base NORDEL values in Table 6.

**Table 6:** **Primary energy factor (MJ-eq/MJ), CO_2_-equivalents, $PO_{4}^{-3}$-equivalents, and SO_2_-equivalents for different electricity mixes (Ecoinvent, 2015)**.

	Base NORDEL	Hard coal electricity mix	Swedish electricity mix
MJ-eq/MJ	2.01	3.24	2.50
g CO_2_-eq/MJ	20	292	11
g ${\text{PO}}_{4}^{-3}$-eq/MJ	0.001	0.027	0.001
g SO_2_-eq/MJ	0.010	0.190	0.011

The sensitivity analysis showed that the hard coal NORDEL increased the input PE of the ammonia route by 53%, while for methanol and DME the increase was on average 50% and for the CHP route the least (43%) (Table 7). The environmental impact categories (GWP, EP, and AP) were all considerably increased by the hard coal electricity mix, as could be expected. The ammonia route showed the highest increase, while the base CHP scenario showed the lowest increase. The increase for DME and methanol was approximately in the same range. A change to the Swedish electricity mix based mainly on nuclear increased the PE by 18, 17, 16, and 14% for ammonia, DME, methanol, and CHP, respectively. The GWP was decreased in the range of 3–7%, with the ammonia route having the greatest reduction and CHP the smallest. The AP decreased on average by 4% for all routes and the EP showed no significant increase for the choice of the Swedish electricity mix.

**Table 7:** **Change (%) in primary energy input and environmental impacts (GWP, EP, and AP) per functional unit when selected input parameters were changed.^a^**

Sensitivity analysis	DME	Methanol	Ammonia	CHP
**Hard coal electricity mix**				
Primary energy	50	50	53	43
GWP	362	337	433	219
EP	40	36	55	23
AP	1,433	1,328	1,624	979
**Swedish electricity mix**				
Primary energy	17	16	18	14
GWP	−6	−5	−7	−3
AP	−4	−4	−4	−3
**Higher range N_2_O emissions factor**				
GWP	112	114	97	119
**Lower range N_2_O emissions factor**				
GWP	−17	−17	−14	−18
**Improved upgrading technology**				
GWP	−17	−17	−14	−18

*^a^“+” indicates increase (%) and “–” indicates decrease (%) in environmental impact as a result of the change in relation to default values*.

Nitrous oxide emissions dominated the GHG emissions from cultivation operations, and were important for all biomass-based pathways. The N_2_O emissions comprised the largest share of emissions from the cultivation unit (unit I), accounting for 73%. According to the IPCC (2006) guidelines, some of the uncertainty in relation to N_2_O emissions from managed soils derives from uncertainty associated with the emissions factor. Higher values within the reported range for N_2_O emissions factors increased the GWP of all routes (by 91–119%), while lower values within the range decreased it (by 14–18%) (Table 7). The methane emissions from the AD and biogas upgrading process contributed considerably to the GWP in the biomass-to-biomethane chain. By optimizing the upgrading technology and improving the biogas management at the plant, it is likely that the emissions can be decreased. A methane loss of 0.5% of the biomethane produced was tested in the sensitivity analysis. However, the results indicated that this would not cause a considerable total reduction for the bio-based routes studied (Table 7).

#### Sensitivity Analysis and Effects on Fossil Replacement

The results from the sensitivity analysis of the bio-based routes were used to evaluate the impact on the net emissions compared with the corresponding fossil products (Table 8). The hard coal electricity mix contributed to a great increase in all environmental impact categories, impairing the performance of all bio-based routes and leading to higher emissions than for the fossil alternatives. The ammonia route had the highest increase, due to high electricity use, and the CHP route the lowest. Even when including combustion of fossil methanol and DME, the bio-based routes resulted in higher GWP than the fossil alternatives. A change to the Swedish electricity mix slightly improved the performance of the bio-based routes, with a subsequent slight reduction in the emissions.

**Table 8:** **Effects of different changes made in the sensitivity analysis on the emissions reduction potential of the different bio-based routes studied**.

	GWP (ton CO_2_-eq/FU)				EP (kg $PO_{4}^{3-}$-eq/FU)				AP (kg SO_2_-eq/FU)			
	DME	Methanol	Ammonia	CHP	DME	Methanol	Ammonia	CHP	DME	Methanol	Ammonia	CHP
Default	−0.6 (−7.3)	1.4 (−5.3)	−2.3	−6.7	16.2	16.9	16.6	16.4	−29.9	−18.0	−6.4	−21.3
Hard coal electricity mix	15.6 (8.9)	16.3 (9.6)	20.3	2.5	23.1	23.1	26.2	20.3	102.5	103.7	177.7	53.8
Swedish electricity mix	−0.9 (−7.6)	1.1 (−5.5)	−2.6	−6.8	16.2	16.8	16.6	16.4	−30.2	−18.4	−6.8	−21.5
Higher range N_2_O emissions factor	4.4 (−2.3)	6.4 (−0.3)	2.8	−1.6	16.2	16.8	16.6	16.4	−29.8	−18.0	−6.35	−21.3
Lower range N_2_O emissions factor	−1.4 (−8.1)	0.6 (−6.1)	−3.0	−7.4	16.2	16.8	16.6	16.4	−29.8	−18.0	−6.3	−21.3
Improved upgrading technology	−1.4 (−8.1)	0.6 (−6.1)	−3.0	−7.4	16.2	16.8	16.6	16.4	−29.8	−18.0	−6.3	−21.3

*Figures in brackets include combustion of fuels*.

An increase in the biogenic N_2_O emissions factor increased the GWP for all bio-based routes. This changed the ammonia route from having a net reduction in GWP to having a higher impact than the fossil alternative (net value 2.8 ton CO_2_-eq/FU). The base CHP scenario was then the only route with a negative net GWP (−1.6 ton CO_2_-eq/FU). However, the bio-based DME route still reduced the GWP in comparison with the fossil alternative (−2.3 ton CO_2_-eq/FU) when combustion of DME was included. Assuming a lower N_2_O emissions factor and reduced methane emissions from the biogas upgrading slightly reduced the net GWP emissions.

## Discussion

Comparison of the different bio-based routes assessed in this study with an input-based FU (1 ha of maize cultivation per year) revealed that the base CHP route had the best performance regarding energy balance and environmental impact categories. PE input and environmental impacts from CHP production were closely related to the biomass-to-biomethane unit, with a low contribution from the CHP unit itself. This indicates that CHP is a highly efficient route for conversion of biogas to alternative energy carriers (Fusi et al., 2016). Among the novel routes assessed, DME and methanol showed better performance regarding energy balance and environmental impact categories than ammonia. However, in this comparison, the differences in product output and the use phase of the products were not considered. When extending the scope to include the substitution of fossil fuels, this becomes a problem since emissions of CO_2_ in the fossil fuel comparators occur in different stages. For fossil-based ammonia and CHP, all CO_2_ emissions occur in the production phase. For fossil DME and methanol (if used as engine fuels) most CO_2_ emissions occur in the use phase. To make a fair comparison, we, therefore, added the CO_2_ emissions for combustion of fossil-based DME and methanol in Table 5.

In Table 5, the emissions from combustion of the bio-based alternatives are accounted as carbon neutral. Unlike bio-based products from forest products, agricultural bio-based products have a short time between the emission from fuel and uptake of the same amount of carbon by new crops and therefore will have very limited or no climate impact (Elshout et al., 2015).

Considering this, it was evident that all bio-based scenarios reduced GWP and AP compared with fossil fuels, while the EP was not considerably affected. It was also clear that the ammonia scenario had a much lower potential in reduction of GWP and AP than the other scenarios. The explanations for this can be many. First of all, the data from fossil alternatives were collected from different literature sources with different assumptions, as it was difficult to find LCA studies with comparable assumptions. Furthermore, in modeling the bio-based alternatives, we also used different data found in the literature, which are comparable in production size but have some differences in assumptions. One such difference is that the DME and methanol are produced in micro-channel reactors with high efficiency at small scale (Hu et al., 2005; LeViness et al., 2011). The ammonia scenario, on the other hand, is modeled using conventional technology scaled down to fit the bio-based production system (Tunå et al., 2014), with lower efficiency at small scale.

The biomass-to biomethane production chain is in general the largest contributor to the PE input and the environmental impact categories in the bio-based routes assessed. The AD and upgrading of biogas to biomethane made a large contribution to the PE input and GWP. The water scrubber is the dominant upgrading technology used during the past decade, with 40% of market share (Bauer et al., 2013). In this study, a water scrubber with a specific power consumption of ~0.2 kWh/Nm^3^ (Bauer et al., 2013) was assumed as the upgrading technology. An option to reduce the PE input could be to choose an amine scrubber, which has a significantly lower electricity demand, about 0.12–0.14 kWh/Nm^3^ depending on plant size, but amine scrubbers also have an external heat requirement for regeneration of the amine solution of about 0.55 kWh/Nm^3^ (Bauer et al., 2013). The heat is most commonly supplied by combusting a part of the raw biogas.

The CH_4_ loss, set to 2% during upgrading of biogas, was the major contributor to GWP in the biomass-to-biomethane production chain. Methane loss levels vary between different technologies, depending on the different process mechanisms. In water scrubbers, some CH_4_ is absorbed into the process water. Most of this is recovered in the flushing tank and is sent back to the gas inlet, but some is lost in the water regenerating step. The results from the sensitivity analysis showed that a reduction in the methane slip to 0.5% of the biomethane produced would reduce the GWP by 14–18% depending on the utilization route. An amine scrubber has methane losses of ~0.1% (Starr et al., 2012) and could be an option to further reduce the GWP.

Cultivation operations for biomass production from maize crops are the largest contributor to EP and the second largest source of PE input and GWP. The high PE input is mainly related to diesel combustion in agricultural machinery. According to Melander et al. (2005), maize cultivation requires high mechanical and chemical inputs, especially in harvesting operations and to combat related weeds, fungi, and pests. A common set of weeds, arthropod pests, and fungal diseases are responsible for the main problems in maize cropping in most European regions, although some differences exist, particularly between northern and southern regions. Options to reduce the high energy and material input into the maize agro-ecosystem include careful choice of variety, cultural control measures, biological control, optimization of pesticide application techniques, and development of more specific control treatments (Melander et al., 2005; Meissle et al., 2010).

The GWP related to maize cropping is mainly related to N_2_O biogenic emissions from soil. However, the levels of biogenic N_2_O emissions from soil are uncertain, as they are mainly influenced by local parameters (Lund et al., 2016), although the most crucial parameter is the level of available nitrogen in soil. The sensitivity analysis showed that increasing the emission factors to higher ranges considerably affected the GWP in all routes. Precision fertilization and other practices for improved efficiency of plant nitrogen uptake can lead to lower N_2_O emissions and also reduced N leaching, which will also result in lower EP (Sogbedji et al., 2000).

Nutrient losses during cultivation of maize crops are the major contributor to EP. Studies by Zhou and Butterbach-Bahl (2014), Perego et al. (2012), and Daudén and Quılez (2004) have revealed a linear relationship between ${\text{NO}}_{3}^{-}$ leaching losses and nitrogen application rates for maize. Improving crop nutrient utilization efficiency (NUE) and reducing the nitrogen surplus are the main strategies for improving agricultural nitrogen management related to ${\text{NO}}_{3}^{-}$ leaching losses. According to Zhou and Butterbach-Bahl (2014), appropriate agricultural nitrogen management practices not only result in near-optimal crop yields but also significantly reduce ${\text{NO}}_{3}^{-}$ leaching losses per kg crop product to a minimum.

Management of digestate is an important way to minimize emissions from the biomass production unit. Management strategies, such as shallow injection of liquid digestate, reduce nitrogen losses in the form of ammonia emissions. Storage of digestate is also important, with many studies showing that open-air digestate storage leads to high acidification and EP through ammonia losses (Rodhe and Nordberg, 2011; Anderson-Glenna and Morken, 2013). Additionally, methane emissions during storage contribute to global warming and other environmental impacts, such as photochemical oxidant creation potential (POCP). These impacts could be reduced by storing the digestate in covered tanks and capturing methane and ammonia (Whiting and Azapagic, 2014).

The sensitivity analysis of electricity mix had the highest effects on the results. With a hard coal electricity mix, the biomethane-based routes had the highest environmental impacts, even compared with fossil alternatives. As expected, this shows that the choice of electricity mix has a considerable impact on the results and that it could be more important than the choice of a specific utilization route for the biomethane produced.

Based on the study presented above, it can be concluded that production of ammonia from non-fossil sources is possible but not competitive with the alternative biomethane-based CHP, DME, and methanol and the fossil-based substitutes. Based on Tunå et al. (2014), this can be explained by economy-of-scale effects, while benefits of non-fossil-based production of ammonia could be security of supply and lower transportation costs.

Overall, the results from this study indicate that production of DME and methanol from biogas could be a feasible alternative regarding energy balance and environmental impact for the enhancement of biogas production and utilization. Furthermore, our previous study (Moghaddam et al., 2015) showed that methanol and DME would be a better choice than compressed biogas in order to reach a national market. The economic feasibility is of course important and is more likely to be a strong constraining factor for biomethane use for methanol and DME production, besides the aspects of energy balance and environmental impact. A Danish technical and economic assessment of a biogas plant for producing steam-reformed methanol of the same magnitude as the plant in our study (~100 GWh/year and 13 500 ton of methanol/year) indicated that the production costs were in the same range as the market price (Pedersen et al., 2014). While that study was based on modeling and various assumptions, which should be considered with care, the aggregated outcome provides incentives for further technical development and studies.

For future LCA studies, the use phase of the different biomethane-based products in the production chain as fuel for agriculture machinery and transportation (DME and methanol) and ammonia as fertilizer input to biomass production could be considered. Furthermore, in this study, we assumed that the biogas for the alternative products was sourced from an existing biogas plant supplied from a cropping system with a steady-state soil carbon level, assuming that there was no land use change involved. With the ambitious political goals of expanding biofuel production, increased demand could in fact lead to pressure on land, causing changes in land use directly and indirectly connected to the bioenergy production. Other studies have shown that land use change can have a large impact on the climate impact of biofuels [see, e.g., review of studies in Ahlgren and Di Lucia (2014)]. Moreover, other environmental impact categories such as ecotoxicity potential due to the emissions of pesticide used for maize cultivation and POCP, which could be related to the emissions of methane from the digestate and the methane losses from the AD plant (Fusi et al., 2016), would be of interest in future studies.

Finally, life cycle cost assessments (LCCAs) would be needed to further provide a better decision support for future technology development and implementation.

## Author Contributions

ÅN and SA supervised the work; EM collected the data, carried out the LCA study, and wrote the paper, with contributions from ÅN and SA.

## Conflict of Interest Statement

The authors declare that the research was conducted in the absence of any commercial or financial relationships that could be construed as a potential conflict of interest.
