# Supplementary material for: Assessment of Novel Routes of Biomethane Utilization in a Life Cycle Perspective
Source: Front Bioeng Biotechnol. 2016 Dec 19;4:89. doi: 10.3389/fbioe.2016.00089 (PMC5165279; doi:10.3389/fbioe.2016.00089)
Supplement: Supplementary file 1 [file table_1.docx]

Table S1. Primary energy input and environmental impacts (GWP, EP, and AP) of biomass-to-biomethane production

|  | Primary energy input (MJ) | GWP  (kgCO_2_ eq.) | EP  (kg PO_4_^3-^ eq.) | AP  (kg SO_2_ eq.) |
| --- | --- | --- | --- | --- |
| *Unit I. Agricultural operations* |  |  |  |  |
| Seedling production^a^ | 144 | 2 | 0.00 | 0.02 |
| Ploughing^b^ | 872 | 59 | 0.07 | 0.53 |
| Harrowing and sowing^b^ | 468 | 32 | 0.04 | 0.29 |
| Weed control^b^ | 149 | 10 | 0.01 | 0.09 |
| Harvest^b^ | 1365 | 93 | 0.11 | 0.84 |
| Harvest transport^b^ | 364 | 25 | 0.03 | 0.22 |
| Silo compaction^b^ | 279 | 19 | 0.02 | 0.17 |
| Fertilizer spreading^b^ | 61 | 4 | 0.01 | 0.04 |
| Liquid digestate spreading^c^ | 209 | 14 | 0.02 | 0.13 |
| Solid digestate spreading^c^ | 264 | 18 | 0.02 | 0.16 |
| Nutrient losses^d^ |  |  | 16.24 |  |
| Biogenic N_2_O emissions^e^ |  | 967 |  |  |
| Nitrogen fertilizer production^d^ | 225 | 32 | 0.01 | 0.05 |
| Phosphorus fertilizer production^d^ | 100 | 13 | 0.01 | 0.21 |
| Potassium fertilizer production^d^ | 430 | 41 | 0.03 | 0.67 |
| *Total agricultural operations* | 4930 | 1328 | 16.62 | 3.41 |
| *Unit II. Road transportation* |  |  |  |  |
| Transport to central digester (harvest)^f^ | 1245 | 85 | 0.10 | 0.76 |
| Transport from central digester (digestate)^f^ | 1594 | 108 | 0.13 | 0.98 |
| Total transportation | 2840 | 193 | 0.24 | 1.74 |
| *Unit III. Biogas production and upgrading* |  |  |  |  |
| Anaerobic digestion plant ^g,h^ | 8642 | 842 | 0.05 | 0.50 |
| Upgrading plant^i^ | 11163 | 1184 | 0.06 | 0.65 |
| Total biogas production and upgrading | 19805 | 2025 | 0.11 | 1.14 |
| *Unit IV. Digestate handling* |  |  |  |  |
| Stirring of digestate^j^ | 100 | 2 | 0.00054 | 0.00575 |
| Pumping of digestate^j^ | 124 | 2 | 0.00068 | 0.00719 |
| Screw press^j^ | 274 | 74 | 0.03246 | 0.08462 |
| Chemical addition^j^ |  | 62 | 0.01069 | 0.02925 |
| Storage (solid digestate)^k,l^ |  | 401 | 0.17690 | 0.00000 |
| Storage (liquid digestate)^l,m^ |  | 122 | 0.00000 | 1.25000 |
| Total digestate handling | 498 | 663 | 0.22 | 1.38 |
| Total | 28073 | 4210 | 17 | 8 |

a. Gissén el al. (2014)

b. Gerin el al. (2008)

c. Whitning & Azapagic (2014)

d. Börjesson et al. (2010)

e. Calculated based on IPCC (2006) methodology

f. Transport of harvest material from farm to central digester (Anaerobic digestion plant_ biogas plant) and transport of the digestate to farm with unloaded returns including loading operation. Transport distance of 20 km assumed, based on Nilson (1995).

g. Pöschl et al. (2010)

h. Berglund & Börjesson ( 2006)

i. Moghaddam et al. (2015)

j. Karunanithi (2014)

k. Anderson-Glenna & Morken (2013)

l. Rodh & Nordberg ( 2013)

m. Wood & Cowie (2014)
