# Supplementary material for: Assessment of Novel Routes of Biomethane Utilization in a Life Cycle Perspective
Source: Front Bioeng Biotechnol. 2016 Dec 19;4:89. doi: 10.3389/fbioe.2016.00089 (PMC5165279; doi:10.3389/fbioe.2016.00089)
Supplement: Supplementary file 2 [file table_2.docx]

**Table S2**.Fertilization regime and digestate application (Börjesson et al., 2010)

| Fertilization regime | | |
| --- | --- | --- |
|  | | |
| N-mineral | kg/ha/yr | 5 |
| P-mineral | kg/ha/yr | 4 |
| K-mineral | kg/ha/yr | 86 |
| Digestate | m^3^/ha/yr | 43 |
| N-digestate | kg/ha/yr | 144 |
| P-digestate | kg/ha/yr | 22 |
| K-digestate | kg/ha/yr | 33 |
