# Supplementary material for: Assessment of Novel Routes of Biomethane Utilization in a Life Cycle Perspective
Source: Front Bioeng Biotechnol. 2016 Dec 19;4:89. doi: 10.3389/fbioe.2016.00089 (PMC5165279; doi:10.3389/fbioe.2016.00089)
Supplement: Supplementary file 3 [file table_3.docx]

**Table S3**. Energy and emissions related to the production process of DME, methanol, ammonia, and CHP per functional unit (only the production process, i.e., excluding biomass-to-biomethane production)

|  | Primary energy input | GWP | EP | AP |
| --- | --- | --- | --- | --- |
|  | GJ | kg CO_2_ eq. | kg PO_4_ ^3-^ eq. | kg SO_2_ eq. |
| DME ^a^ | 15 | 266.31 | 0.21 | 1.56 |
| Methanol ^a^ | 13 | 216.77 | 0.19 | 1.49 |
| Ammonia ^b^ | 29 | 998.37 | 0.34 | 3.66 |
| CHP ^c.d^ | 1 | na* | na | na |

* na; not available

a. Moghaddam et al. (2015)

b. Makhlouf et al. (2015)

c. Goehner et al. (2013)

d. Ecoinvent (2015)
