# Supplementary material for: Assessment of Novel Routes of Biomethane Utilization in a Life Cycle Perspective
Source: Front Bioeng Biotechnol. 2016 Dec 19;4:89. doi: 10.3389/fbioe.2016.00089 (PMC5165279; doi:10.3389/fbioe.2016.00089)
Supplement: Supplementary file 4 [file table_4.docx]

**Table S4.** Emissions factors for fossil substitutes in different routes studied (Ecoinvent, 2015)

|  | | | |
| --- | --- | --- | --- |
|  | *kg CO_2_ eq./kg fuel* | *kg NO_x_ eq./kg fuel* | *kg SO_2_ eq./kg fuel* |
| DME^a^ | 1.15 | 2.10E-03 | 7.70E-03 |
| Methanol^a^ | 0.53 | 8.00E-04 | 4.50E-03 |
|  | *kg CO2 eq./MJ* | *kg NOx eq./MJ* | *kg SO2 eq./MJ* |
| Heat^b^ | 0.02 | 1.70E-05 | 6.50E-05 |
| Electricity^b^ | 0.14 | 8.33E-05 | 3.33E-04 |
| Steam^c^ | 0.02 | 4.57E-05 | 1.43E-04 |
|  | *kg CO2 eq./kg ammonia* | kg NO_x_ eq./kg ammonia | *kg SO2 eq./kg ammonia* |
| Ammonia^d^ | 2.0 | 0.0019 | 0.003 |

a. DME and methanol from natural gas.

b. Heat and power co-generation based on natural gas in a combined cycle power plant.

c. Steam production in fossil-based chemical industry

d. Ammonia production from natural gas
