# Supplementary material for: Assessment of Novel Routes of Biomethane Utilization in a Life Cycle Perspective
Source: Front Bioeng Biotechnol. 2016 Dec 19;4:89. doi: 10.3389/fbioe.2016.00089 (PMC5165279; doi:10.3389/fbioe.2016.00089)
Supplement: Supplementary file 5 [file presentation_1.pdf]

References for Tables in Supplementary Material

Anderson-Glenna, M., & Morken, J. (2013). Greenhouse gas emissions from on-farm digestate storage facilities. *Tel-Tek report*, (2213040-1).

Berglund, M., & Börjesson, P. (2006). Assessment of energy performance in the life-cycle of biogas production. *Biomass and Bioenergy, 30*(3), 254-266.

Börjesson, P., Tufvesson, L., & Lantz, M. (2010). Life cycle assessment of biofuels in Sweden. *Environmental and Energy System Studies report no. 70.*

Ecoinvent, Swiss Centre for Life Cycle Inventories. (2015).

Gerin, P. A., Vliegen, F., & Jossart, J. M. (2008). Energy and CO 2 balance of maize and grass as energy crops for anaerobic digestion. *Bioresource Technology, 99*(7), 2620-2627.

Gissén, C., Prade, T., Kreuger, E., Nges, I. A., Rosenqvist, H., Svensson, S. E., ... & Björnsson, L. (2014). Comparing energy crops for biogas production–yields, energy input and costs in cultivation using digestate and mineral fertilization. *Biomass and bioenergy*, *64*, 199-210.

Goehner, A., Wong, R. & McCulloch, M. (2013). LNG for Yukon Energy Power Generation, A Life Cycle Emissions Inventory. *The Pembina Institute.*

Karunanithi. A. (2014). System analysis of de-watering process for treating biogas digestate. Linköping University, Sweden

Makhlouf, A., Serradj, T., & Cheniti, H. (2015). Life cycle impact assessment of ammonia production in Algeria: A comparison with previous studies. *Environmental Impact Assessment Review*, *50*, 35-41.

Moghaddam, E. A., Ahlgren, S., Hulteberg, C., & Nordberg, Å. (2015). Energy balance and global warming potential of biogas-based fuels from a life cycle perspective. *Fuel Processing Technology*, *132*, 74-82.

Nilsson, D. (1995). Transportation work and energy requirements for haulage of straw fuels. A comparison between the plants at Såtenäs and Svalöv. *Swedish Journal of Agricultural Research*, *25*(3), 137-141

Pöschl, M., Ward, S., & Owende, P. (2010). Evaluation of energy efficiency of various biogas production and utilization pathways. *Applied Energy*, *87*(11), 3305-3321.

Rodhe, L. & Nordberg, Å. (2011) Greenhouse gas emissions from the storage of liquid and solid manure and abatement strategies. *Publishe in*: Emissionen der Tierhaltung. Treibhausgase, Umweltbewertung, Stand det Techik.

Whiting, A., & Azapagic, A. (2014). Life cycle environmental impacts of generating electricity and heat from biogas produced by anaerobic digestion. *Energy*, *70*, 181-193.

Wood, S., & Cowie, A. (2004). A Review of Greenhouse Gas Emission Factors for Fertiliser Production. IEA Bioenergy Task 38. *Cooperative Research Centre for Greenhouse Accounting. Research and Development Division,* State Forests of New South Wales.
